# An analysis of identical single-nucleotide polymorphisms genotyped by two different platforms

**DOI:** 10.1186/1471-2156-6-S1-S152

**Published:** 2005-12-30

**Authors:** Brian K Suarez, Chelsea Taylor, Sarah Bertelsen, Laura J Bierut, Gerald Dunn, Carol H Jin, John SK Kauwe, Andrew D Paterson, Anthony L Hinrichs

**Affiliations:** 1 Department of Psychiatry, Washington University School of Medicine, St., Louis, MO, USA; 2 Department of Genetics, Washington University School of Medicine, St., Louis, MO, USA; 3 Program in Population Health Sciences and Program in Genetics and Genomic Biology, The Hospital for Sick Children, Toronto, Canada; 4 Program in Genetics and Genomic Biology, The Hospital for Sick Children, Toronto, Canada; 5 Department of Public Health Sciences and Psychiatry, University of Toronto, Canada

## Abstract

The overlap of 94 single-nucleotide polymorphisms (SNP) among the 4,720 and 11,120 SNPs contained in the linkage panels of Illumina and Affymetrix, respectively, allows an assessment of the discrepancy rate produced by these two platforms. Although the no-call rate for the Affymetrix platform is approximately 8.6 times greater than for the Illumina platform, when both platforms make a genotypic call, the agreement is an impressive 99.85%. To determine if disputed genotypes can be resolved without sequencing, we studied recombination in the region of the discrepancy for the most discrepant SNP rs958883 (typed by Illumina) and tsc02060848 (typed by Affymetrix). We find that the number of inferred recombinants is substantially higher for the Affymetrix genotypes compared to the Illumina genotypes. We illustrate this with pedigree 10043, in which 3 of 7 versus 0 of 7 offspring must be double recombinants using the genotypes from the Affymetrix and the Illumina platforms, respectively. Of the 36 SNPs with one or more discrepancies, we identified a subset that appears to cluster in families. Some of this clustering may be due to the presence of a second segregating SNP that obliterates a *Xba*I site (the restriction enzyme used in the Affymetrix platform), resulting in a fragment too long (>1,000 bp) to be amplified.

## Background

All markers are not created equal with respect to information content, ease of genotyping, or the frequency of miscalls.

Genotyping errors are a source of concern in both linkage and association analyses. In a linkage study it is generally easier to detect genotyping errors for microsatellites than for single-nucleotide polymorphisms (SNPs) because microsatellite errors are more likely to give rise to Mendelian inheritance incompatibilities. However, because SNPs are primarily diallelic, genotyping errors often do not give rise to a Mendelian transmission inconsistency. Indeed, in some settings such as a linkage study of affected sibships, it is impossible to detect genotyping errors for individual SNP markers if parents are not genotyped.

In this study we compare the genotypic calls reported by two competing SNP platforms: Illumina and Affymetrix. We identified 91 autosomal and 3 X-linked SNPs that are common to both datasets.

## Methods and Results

Because the allele designations are arbitrary, we decided to use the Illumina designations as our template. In the Affymetrix data, 50 of the 94 SNPs used the same allele designations as Illumina. For the remaining 44 SNPs, we relabeled the alleles to achieve comparability.

Table 1 compares the allele calls for the two platforms for the 94 SNPs using all of the Collaborative Study on the Genetics of Alcoholism (COGA) data. The numbers in parentheses are the counts prior to data cleaning. As expected, the number of no-calls increased in the clean data as a result of removing Mendelian incompatibilities. For the 116,457 occurrences in which a genotype call could be made with both platforms, a total of 180 (0.15%) are discrepant. None of these discrepancies gives rise to a Mendelian non-inheritance, in part because of the data cleaning process that selectively removed an "offending" genotype.

The no-call rate is higher for the Affymetrix platform than for the Illumina platform. In the cleaned data for these 94 SNPs, the difference in the no-call rate is approximately 8.6-fold. There is, however, an interesting pattern in the distribution of no-calls. The Affymetrix no-call rate is 3.79% for genotypes that Illumina calls as homozygous and 6.53% for genotypes that Illumina calls heterozygous. The difference in call rates conditioned on Illumina's genotypes is highly significant (*p *< 0.0001). This patterning is not present for Illumina no-calls.

The distribution of the 180 discrepancies is also nonrandom. There are no occurrences of one platform calling a genotype a homozygote and the other platform calling the genotype the opposite homozygote. Rather, all of the discrepancies result from a genotype being called homozygous by one platform and heterozygous by the other. In 86% of the discrepancies, Illumina calls the genotype heterozygous and Affymetrix calls it homozygous.

There are no discrepancies for 58 (62%) of the 94 SNPs (Table 2). For 14 SNPs, there is a single discrepancy. For the remaining 22 SNPs, there are 2 or more discrepancies. For 8 of these 22 SNPs with multiple discrepancies, there is no evidence for family clustering. For the remaining 14 SNPs, however, the discrepancies give evidence of clustering in families. In almost every intra-familial discrepancy (83 out of 88 occurrences), the Illumina assay calls the genotype heterozygous.

Two methods are commonly used to check for the presence of genotyping errors. The first – especially useful in case/control association studies – is to determine if more than the expected number of markers are out of Hardy-Weinberg equilibrium (HWE). The second method, appropriate for family data, is to determine if double recombinants can be inferred over a short genomic region.

We performed a HWE assessment of the 34 autosomal SNPs that showed one or more discrepancies between the two platforms. This assessment was performed on a panel of 245 unrelated Whites drawn from the COGA families. None of the 34 SNPs from the Illumina platform was significantly out of equilibrium. Four of the 34 SNPs from the Affymetrix platform were found not to be in HWE (tsc0546488, *p *< 0.0001; tsc1018661, *p *= 0.014; tsc0039689, *p *= 0.023; tsc0280570, *p *= 0.022). None of these departures from HWE proportions is due to discrepancies in allele calling, however, because the number of discrepancies for tsc0546488, tsc1018661, tsc0039689, and tsc0280570 are only 1, 1, 3, and 1, respectively. Rather, the departures from HWE are due to differential no-calls in the Affymetrix platform. Compared to their Illumina counterparts (where genotypes are reported for all 245 unrelated individuals for each SNP), the number of no-calls for tsc0546488, tsc1018661, tsc0039689, and tsc0280570 are 51, 25, 18, and 28. For the first 3 SNPs all of the no-calls were for genotypes called heterozygous in the Illumina platform; for the fourth SNP, all of the no-calls were for genotypes called 1/1 by Illumina. Accordingly, it is the nonrandomness of the no-calls that leads to the departure from HWE for these 4 Affymetrix SNPs.

**Figure 1:**
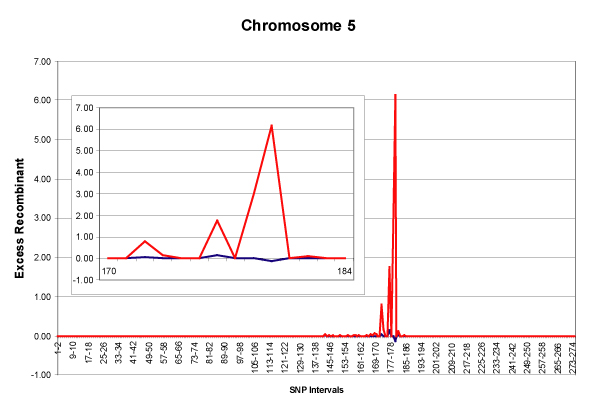
**Distribution of excess recombinants**. The red curve uses the Affymetrix genotype (tsc0260848) at marker 180. The blue curve uses the Illumina genotype (rs958883) at marker 180. The inset shows an enlarged view of the excess number of inferred recombinants in the neighborhood of rs958883/tsc0260848.

We also searched for evidence of multiple recombinants. We limited our analysis to rs958883/tsc0260848 in White families, since this SNP gave the largest number of discrepancies between the two platforms. SNP rs958883 is marker 180 on Illumina's chromosome 5 map. The 12 White families with discrepancies for this SNP contained a total of 186 meioses. We used the COUNT REC option in GENEHUNTER version 2.1 [1] to infer the positions of recombination for all of the markers on this chromosome. We first analyzed all 276 markers in the Illumina set using the genetic map provided by the company. We then substituted the tsc0260848 genotypes from the Affymetrix platform for the rs958883 genotypes and repeated the analysis. Finally, we deleted rs958883/tsc0260848 altogether and repeated the analysis a third time. Figure 1 plots the results obtained by subtracting the inferred number of recombinants in each interval (when the inconsistent marker is removed), from the inferred number of recombinants when the Illumina genotypes (blue curve) or the Affymetrix genotypes (red curve) are included. There is an unmistakable region of increased recombination in the vicinity of the marker 180 when the Affymetrix genotypes are used. Figure 1's inset shows that the largest number of inferred recombinants occurs in the expected interval. Two upstream peaks also occur due to stretches of uninformative markers in some families.

**Figure 2:**
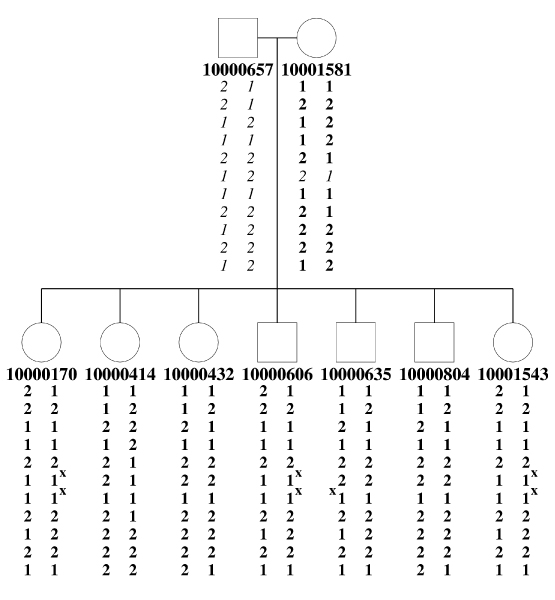
**Inferred haplotypes for markers 175 to 185 for the core part of pedigree 10043**. Italics indicated that genotypes were reconstructed. 'x' marks the interval where a recombinant is inferred to have occurred. The actual double recombinants are probably persons 10000432, 10000635, and 10000804 for reasons given in the text. The physical distance between the SNPs flanking the double recombinants is 1.57 Mb.

While subroutines such as COUNT REC estimate the total number of recombinants in an interval, other subroutines are useful for inferring double recombinants in an individual. We used GENEHUNTER's HAPLOTYPE option and MERLIN's version 0.9.12b [2] best option to inspect the inferred haplotypes in the core nuclear family of pedigree 10043. Individuals 10000432, 10000635, and 10000804 were genotyped as heterozygotes with the Illumina platform and as 2/2 homozygotes with the Affymetrix platform. Figure 2 shows the GENEHUNTER output of the core pedigree for SNP markers 175–185. All genotypes are from Illumina except the middle marker, where the genotypes from Affymetrix have been substituted for rs958883. GENEHUNTER and MERLIN gave identical inferences for this pedigree. Both identified 3 double recombinants in adjacent intervals-namely intervals 179–180 and 180–181. When the Illumina genotypes are used, no double recombinants are inferred by either GENEHUNTER or MERLIN. (NB: in both the raw and the cleaned Illumina data set, the mother's (10001581) genotype at rs958883 is recorded as 1/1 whereas in the raw and the cleaned Affymetrix data set, the mother's genotype at tsc0260848 is recorded as 1/1 and 0/0, respectively.) The identified double recombinants occur in individuals whose genotypes are consistent between the two platforms. There are 2 equally probable inferences: either offspring 10000170, 1000060, and 10001543 (all of whom are genotyped as 1/1 with both platforms) are double recombinants and offspring 10000432, 10000635, and 10000804 (all of whom are genotyped as 2/2 with the Affymetrix platform) are not double recombinants, or vice versa. The evidence suggests that the 2/2 genotypes give rise to the apparent double recombinations.

## Discussion

There is remarkable agreement for SNP genotypes called with the Illumina and Affymetrix platforms. The concordance is estimated to be 99.85%. However, there is a substantially higher no-call rate with the Affymetrix platform and the no-call rate appears to be genotype- and SNP-specific. This differential no-call rate gave rise to 4 (13%) significant departures from HWE in 32 SNPs that showed one or more discrepancy between the two platforms. The SNP that showed the largest number of discrepancies (rs958883 in the Illumina dataset and tsc0260848 in the Affymetrix dataset) was analyzed to determine if the presence of double recombinants could help resolve the differences. Analysis of recombination in 12 White families gave persuasive evidence of increased recombination using the Affymetrix genotypes but no increase using the Illumina genotypes. Analysis of a single family that contained 3 offspring with the disputed genotype identified 3 double recombinants using the Affymetrix genotypes but none with the Illumina genotypes.

The clustering of discrepancies within families is consistent with the hypothesis that there is another SNP or insertion/deletion that obliterates an *Xba*I site in the vicinity of rs958883/tsc026848, thereby resulting in a fragment that is too large to amplify. The closest 5' and 3' *Xba*I sites are located 134 nucleotides upstream and 519 nucleotides downstream. When both of these *Xba*I sites are intact, the resulting fragment is within the size range (250–1,000 bp) that can be amplified using common adapters. For rs958883/tsc0260848, NCBI's dbSNP (build 123) indicates that there is, indeed, another SNP (rs17150546) in the closest upstream *Xba*I site that changes the wild-type sequence (TCTAGA) to TCGAGA. When this *Xba*I site is obliterated, the resulting fragment is 3,617 nucleotides- clearly too long to be amplified. It is perhaps of interest that the SNP that gave the second highest number of discrepancies, rs768224/tsc0075731, also has a SNP (rs10977965) in its neighboring *Xba*I site. When the minor allele is present at this SNP, the resulting fragment is 2,978 nucleotides in length- again, too long to be amplified. Accordingly, these two known SNPs in *Xba*I sites would account for 32% of the 180 observed discrepancies. Of course, a definitive test of this hypothesis would require sequencing discrepant individuals at the respective *Xba*I sites.

## Abbreviations

COGA: Collaborative Study on the Genetics of Alcoholism

HWE: Hardy-Weinberg equilibrium

SNP: Single-nucleotide polymorphism

## Authors' contributions

BKS, CT, and ADP conceived and executed the research. ALH, CT, and ADP identified the SNPs common to both datasets. ALH carried out the HWE analysis and the COUNT REC analysis. JSKK identified the 245 unrelated Whites used in the HWE analysis. CHJ carried out the haplotype analysis of pedigree 10043. BKS drafted the manuscript. SB, LJB, and GD participated in the design of the study and gave critical advice on earlier drafts of the manuscript. All authors have read and approved the final version of this manuscript.

## Figures and Tables

**Table 1:** Joint distribution of genotypes produced by the Illumina and Affymetrix platforms.

Illumina	Affymetrix				
	
	0/0^a^	1/1	1/2	2/2	Total
0/0^a^	106 (55)	183 (181)	261 (257)	176 (172)	726 (665)

1/1	1415 (1395)	31993 (32188)	5 (7)	0 (0)	33413 (33590)

1/2	3562 (3544)	38 (50)	50816 (51098)	116 (137)	54532 (54829)

2/2	1168 (1156)	0 (0)	21 (28)	33468 (33624)	34657 (34808)

Total	6251 (6150)	32214 (32419)	51103 (51390)	33760 (33933)	123328 (123892)

The numbers in parentheses are from the data that include errors due to Mendelian incompatibility.

^a^0/0 denotes missing genotypes (no-calls).

**Table 2:** Frequency distribution for discrepancies in 94 SNPs genotyped in both Illumina and Affymetrix linkage panels.

Number of Discrepancies	Frequency	Familial^a^	Unique^b^
0	58	-	-
1	14	-	-
2	6	2 (2)	8
3	5	1 (3)	12
4	2	1(2),1(4)	2
5	1		5
6	1	1(2)	4
9	2	2(2),1(3)	11
12	1	2(2),2(4)	0
16	1	5(2)	6
17	1	1(2),1(3)	12
21	1	2(2),1(4),1(5),1(6)	2
36	1	4(2),4(3),2(4)	8

^a^The number of different families and the number of persons in the family (in parentheses) that show the discrepancy.

^b^The number of families with just one discrepancy.
